# Supplementary figures and images for: Safety and efficacy of quick-soluble gelatin microparticles for transarterial embolization of the lower urinary tract: Preclinical study in a rabbit urinary bladder embolization model
Source: PLoS One. 2025 Nov 12;20(11):e0335894. doi: 10.1371/journal.pone.0335894 (PMC12611118; doi:10.1371/journal.pone.0335894)

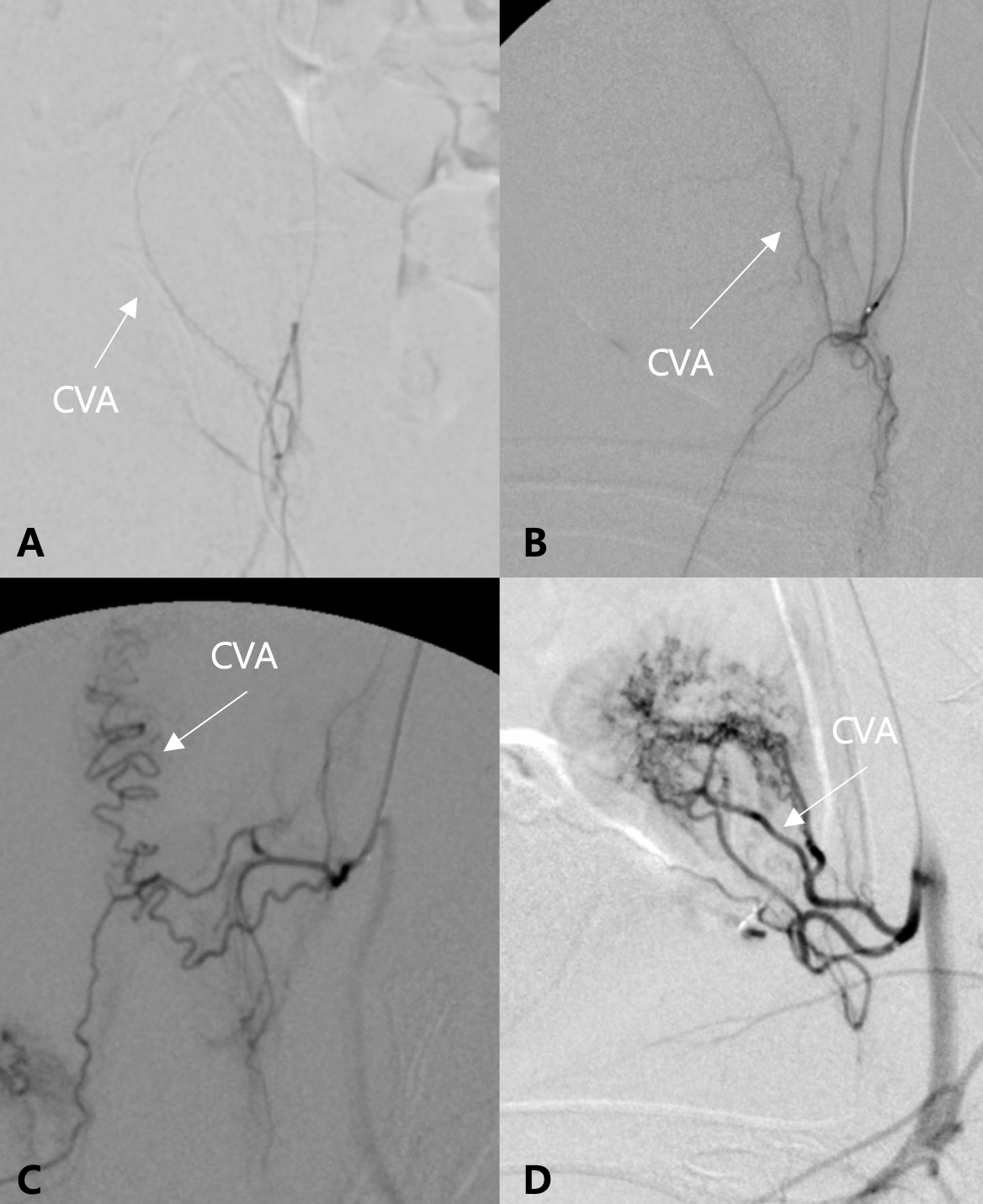

Supplement: S1 Fig — (A) Score 1: Moderate reduction in contrast blush in the bladder wall with cranial vesical artery patency. (B) Score 2: Normal contrast blush in the bladder wall with cranial vesical artery patency. (C) Score 3: Mild hypervascularization in the bladder wall with increased neovascularization. (D) Score 4: Dense contrast blush with marked hypervascularization in the bladder wall. (TIFF) [file pone.0335894.s001.tiff]

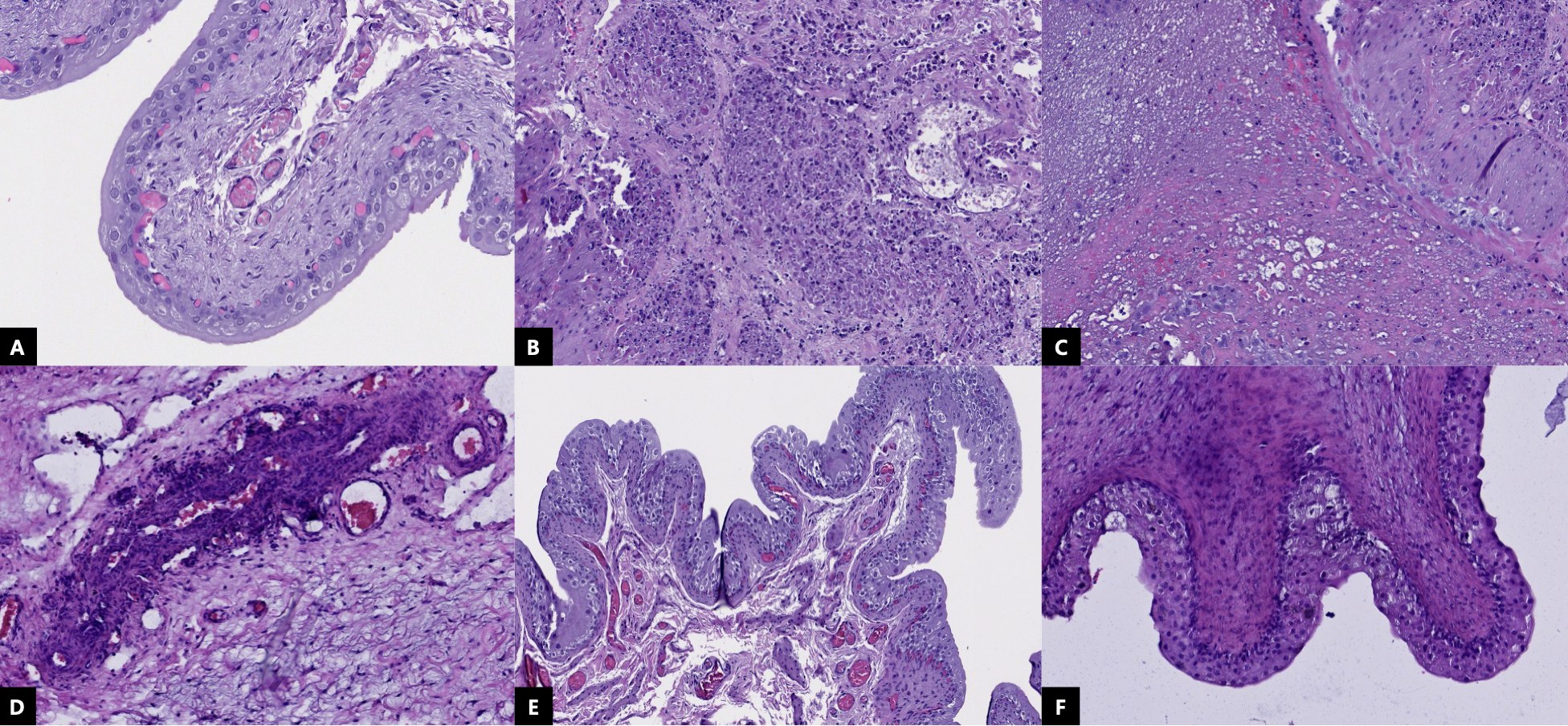

Supplement: S2 Fig — (A) Congestion and edema: Severe congestion observed in the mucosal and submucosal layers of the urinary bladder. (B) Inflammatory cell infiltration: The lamina propria and submucosa show evident vascular structural damage, accompanied by perivascular infiltration of inflammatory cells. (C) Necrosis: Severe necrosis around the vascular damage region. (D) Fibroblast proliferation: Severe infiltration of lymphoid cells was present in the mucosa, submucosa, and muscular layers, accompanied by the deposition of basophilic hyaline-like materials, neovascularization, and fibroblast infiltration (E) Neovascularization: Both neovascularization and the formation of mature blood vessels are evident. (F) Epithelial regeneration: The transitional epithelium of the bladder showed complete recovery. (TIFF) [file pone.0335894.s002.tiff]
