# Supplementary material for: Safety and efficacy of quick-soluble gelatin microparticles for transarterial embolization of the lower urinary tract: Preclinical study in a rabbit urinary bladder embolization model
Source: PLoS One. 2025 Nov 12;20(11):e0335894. doi: 10.1371/journal.pone.0335894 (PMC12611118; doi:10.1371/journal.pone.0335894)
Supplement: S3 Table — (DOCX) [file pone.0335894.s003.docx]

**S3 Table. Body weight of the rabbits on each euthanization day**

| Time-point | Body weight (kg) |
| --- | --- |
| Day 0 | 3.70 ± 0.24 (n = 12) |
| Day 3 | 3.33 ± 0.23 (n = 9) |
| Day 7 | 3.20 ± 0.28 (n = 6) |
| Day 14 | 3.44 ± 0.22 (n = 3) |

Data are presented as means ± standard deviations
