# Supplementary material for: Safety and efficacy of quick-soluble gelatin microparticles for transarterial embolization of the lower urinary tract: Preclinical study in a rabbit urinary bladder embolization model
Source: PLoS One. 2025 Nov 12;20(11):e0335894. doi: 10.1371/journal.pone.0335894 (PMC12611118; doi:10.1371/journal.pone.0335894)
Supplement: S4 Table — (DOCX) [file pone.0335894.s004.docx]

**S4 Table. Rabbits with gross and occult hematuria**

| Day | Number of rabbits with gross hematuria / Total | Number of rabbits with occult hematuria / Total |
| --- | --- | --- |
| 0 | 9 / 9 | 0/12 |
| 1 | 9 / 9 | ND |
| 2 | 6 / 9 | ND |
| 3 | 4 / 9 | 9/9 |
| 4 | 0 / 6 | ND |
| 5 | 0 / 6 | ND |
| 6 | 0 / 6 | ND |
| 7 | 0 / 6 | 6/6 |
| 8 | 0 / 3 | ND |
| 9 | 0 / 3 | ND |
| 10 | 0 / 3 | ND |
| 11 | 0 / 3 | ND |
| 12 | 0 / 3 | ND |
| 13 | 1 / 3 | ND |
| 14 | 0 / 3 | 2/3 |

ND, not done
