# Supplementary material for: Safety and efficacy of quick-soluble gelatin microparticles for transarterial embolization of the lower urinary tract: Preclinical study in a rabbit urinary bladder embolization model
Source: PLoS One. 2025 Nov 12;20(11):e0335894. doi: 10.1371/journal.pone.0335894 (PMC12611118; doi:10.1371/journal.pone.0335894)
Supplement: Attachment — Submitted filename: Response to Reviewer 2.docx [file pone.0335894.s006.docx]

**Response to Reviewer**

**Reviewer #2: General Comments**

C: This manuscript investigates the safety and efficacy of quick-soluble gelatin microparticles (QS-GMP) in a rabbit urinary bladder embolization model. The study is methodologically well-structured, with comprehensive evaluation using angiography, histopathology, hematological and biochemical analyses. The establishment of a reproducible preclinical bladder embolization model is of clear significance and could contribute to the development of safer embolization strategies for lower urinary tract interventions.　However, there are several issues that need to be addressed before publication. The writing sometimes overstates the novelty or clinical readiness of QS-GMP, whereas the data mainly support feasibility and preliminary safety. Furthermore, while angiographic and histopathological scoring systems are well-described, representative images illustrating these criteria should be included to enhance clarity and reproducibility. Finally, the Discussion could be improved by sharpening the interpretation of findings, avoiding overstatements, and more clearly positioning the contribution of this model within the broader field of embolization research.

**R:** We sincerely appreciate the reviewer’s constructive and encouraging feedback. We have addressed the points you mentioned and made the necessary revisions to our manuscript. According to your suggestions, we have toned down overstatements in the manuscript. In addition, we supplemented the manuscript with representative angiographic and histopathological images to support the scoring systems in the tables.

**Abstract**

**C:** Line 20: The phrase “promising embolic agents” is overstated at this stage. Since QS-GMP are still in early evaluation, “emerging embolic agents” or “novel embolic agents under investigation” would be more appropriate.

**R:** Thank you for pointing this out. We have replaced the phrase “promising embolic agents” with “emerging embolic agents”.

**Discussion**

**Paragraph 1**

**C:** While the rationale is clear, the paragraph could be strengthened by more explicitly comparing this model to other existing embolization models (e.g., hepatic, renal, prostatic).

**R:** Thank you for the suggestion. We have revised Paragraph 1 of the Discussion to include comparisons with hepatic, renal, and prostatic embolization models.

**Paragraph 5**

**C:** The discussion of transient hypervascularization is interesting but could be expanded by clarifying whether this represents reactive angiogenesis or an artifact of inflammation. The comparison to hepatic TAE studies is valuable, but it should be noted that urinary bladder physiology may differ substantially.

**R:** Thank you for this insightful suggestion. We have revised the paragraph to clarify that the observed hypervascularization may reflect either reactive angiogenesis or inflammatory hyperemia, and we have acknowledged that bladder physiology differs from hepatic circulation, which may influence the vascular response.

**Paragraph 6**

**C:** The safety profile is presented convincingly, but the conclusion is slightly overstated. The data support feasibility and short-term safety, but not necessarily long-term safety or clinical application. The language should be moderated accordingly.

**R:** As noted in our general response, we have toned down the statements in the Abstract, Discussion, and Conclusion to reflect feasibility and short-term safety.

**C:** Table 1 and Table 4: The scoring system for angiographic findings is clearly described, but representative angiographic images corresponding to each score should be provided. This will help readers understand how the criteria were applied in practice.

Table 2: Similarly, representative histopathological images illustrating each scoring category (edema, necrosis, inflammation, neovascularization, regeneration, etc.) should be included. Without these, it is difficult to validate the scoring method.

**R:** Thank you for the helpful suggestion. In accordance with the journal’s figure limit, we have added representative angiographic images as Supplementary Figures. These include examples corresponding to each angiographic score category (Tables 1 and 4) and each histopathological scoring parameter (Table 2).
